# Supplementary material for: Are carbon emissions trading and green financial instruments synergistic? -Comprehensive quantitative research based on content analysis
Source: PLoS One. 2024 Mar 7;19(3):e0298601. doi: 10.1371/journal.pone.0298601 (PMC10919601; doi:10.1371/journal.pone.0298601)
Supplement: S1 Data — (ZIP) [file pone.0298601.s001.zip › Data/地级市Z_F工作报告.zip!/┴╔─■/╔≥╤⌠╩╨/└┤╫╘╣1⁄2╓┌║┼í╛┬φ┐╦╩2╛▌═°í┐_2008.docx]

各位代表：

　　现在，我代表本届政府向大会作工作报告，请予审议，并请市政协委员提出意见和建议。

　　一、过去五年政府工作回顾

　　五年来，在省委、省政府和市委的正确领导下，市政府带领全市人民，紧紧抓住中央实施振兴东北等老工业基地战略的重大机遇，深入贯彻落实科学发展观，坚持工业立市，开拓进取，奋力拼搏，全面完成了市第十三届人民代表大会所确定的各项任务，改革开放和社会发展取得新的重大成就，沈阳老工业基地由调整改造进入了全面振兴的新阶段。

　　2007年，全市经济社会实现又好又快发展，呈现出经济增长、结构优化、效益提高、民生改善的良好势头，主要经济社会指标大幅提升，城市综合实力明显增强。

　　——预计全市地区生产总值实现3055亿元，比上年增长17.7%，增幅为近15年来最高水平，比2002年增长1.1倍；

　　——全社会固定资产投资完成2327亿元，比上年增长30%，比2002年增长4.8倍；

　　——社会消费品零售总额实现1229亿元，比上年增长17.2%，为近10年来最高增幅，比2002年增长90%；

　　——实际利用外资实现50.4亿美元，创历史新高，比上年增长66%，比2002年增长2.4倍；

　　——地方财政一般预算收入完成230.1亿元，比上年增长30.9%，比2002年增长2.6倍；

　　——城市居民人均可支配收入达到14600元，比上年增长25.3%，是近12年来增长最快的年份，比2002年增长1.1倍；

　　——农民人均纯收入达到7080元，比上年增长24%，比2002年增长1倍；

　　——城镇登记失业率为3.35%，是近7年来最低点，首次低于全国和全省的平均水平；

　　——单位地区生产总值能耗比上年下降5.1%，主要污染物排放总量比上年减少3%。

　　经济结构战略性调整取得重大突破。五年来，在加快推进体制机制创新的同时，着力调整优化产业结构，促进了经济的快发展、大发展。

　　工业持续快速增长。2007年规模以上工业增加值实现1220亿元，比上年增长31%，比2002年增长3.6倍。工业运行质量和效益显著提高，规模以上工业实现利润145.7亿元，比上年增长95.1%。通过一手抓项目、一手抓空间建设，沈西工业走廊、沈北新区及重点工业园区发展迅猛，“东汽、西重、南高、北农”新的产业发展格局初步形成。装备制造业增势强劲，规模以上增加值占全市工业的比重达到47.5%。远大、北方重工等6户骨干企业和铁西汽车零部件等4大产业集群实现百亿产出。沈阳机床集团荣获中国工业大奖表彰奖。百万吨乙烯压缩机组、大型盾构机、燃气轮机等重大装备国产化实现突破。全市工业由举步维艰到生机勃发、重新崛起，沈阳正在由工业大市向工业强市转变。

　　服务业呈现较快发展态势。2007年全市服务业增加值完成1451亿元，比上年增长14%，占地区生产总值比重达到47.5%。连锁经营、代理、电子商务等现代流通方式不断涌现，大型超市、购物中心等新兴业态蓬勃发展，物流、金融、信息、动漫等现代服务业成长壮大，现代服务业比重明显提高，服务业结构不断优化。金融区域服务能力增强，商业银行成功重组为盛京银行，实现跨区域经营，外资银行分行及代表处达到9家。房地产市场健康平稳发展，旅游总收入年均增长17.8%，会展交易额比2002年增长近2倍。恒隆市府广场、华润万象城等78个重大项目相继开工建设，都市中央走廊初见形象。

　　高新技术产业快速发展。2007年全市高新技术产值实现2100亿元，比上年增长40%，比2002年增长2.4倍。浑南新区示范和聚集作用不断增强，建设了富士康科技园、中科院沈阳科技创新园、自动识别技术产业园等一批重大科技创新产业基地。高新技术产业领域不断扩展，IC装备、软件等产业初具规模，数字医疗、工业机器人等制造业高端产品已实现产业化。自主创新能力不断增强，45项科技成果荣获国家级科技奖，发明专利申请量比2002年增长2.6倍；研发、生产了百万伏特高压电抗器、兆瓦级风力发电机组等一批具有自主知识产权和国际领先水平的重大产品。

　　县域经济活力明显增强。2007年县域地区生产总值比上年增长21.5％，占全市地区生产总值比重达到36.9%。地方财政一般预算收入比上年增长60.2%，比2002年增长4倍。粮食产量创历史新高。规模以上工业增加值占全市工业比重达到48%，已成为县域经济发展的主导力量。现代农业发展迅速，高效特色农业种植面积比2002年翻一番，畜牧业产值占农业总产值比重达到50%，农产品加工率达到49%，沈北新区已成为全国最大的农产品深加工基地。

　　改革开放取得突破性进展。五年来，坚持以改革开放促调整、促改造、促发展，老工业基地振兴的内在动力不断增强。

　　全市加快推进债务重组，实现企业减债137.7亿元。24户国有重点企业股份制改造基本完成，国有中小企业全部完成改制任务，沈阳机床、北方重工成功实现国际并购。企业融资渠道不断拓宽，全市新增上市公司10户。铁西区企业整体搬迁改造全面完成，全区面貌发生了脱胎换骨的变化，被国家发改委和振兴东北办授予“老工业基地调整改造暨装备制造业发展示范区”称号。产权、土地、金融、技术、人才等要素市场体系进一步健全，政策、法规逐渐完善，市场秩序走向规范。行政体制改革不断深化，市级审批事项减少到110项，“即办件”比率达到90%以上。非公有制经济不断发展壮大，2007年完成增加值占全市地区生产总值的64%，已成为推动全市经济发展的主导力量。

　　对外开放实现战略升级。招商引资取得显著成果，五年累计实际利用外资148.6亿美元，调入内资1754亿元。引进超千万美元重大外资项目279个，51家世界500强企业来沈投资兴业。对外经贸稳步发展，进出口总额累计实现265亿美元，新批境外投资项目43个。对外交往日益活跃，法国、爱尔兰等10个国家的元首、政府首脑来沈访问。新开通国际航线34条，进出空港人数增长1.2倍，接待入境游客162.4万人次。世园会、制博会、世遗会、世界生产力大会、东北亚区域发展与合作论坛等重大国际活动取得巨大成功，极大地提高了沈阳的国际知名度和影响力。

　　城市环境质量明显改善。五年来，旧城综合改造任务基本完成，市容环境综合整治成效显著，城市建设和管理进入到全面提升质量的新阶段。2007年城建投资完成207亿元，比上年增长33.2%，五年累计投资662亿元，年均增长28.5%。

　　规划建设了大浑南地区、西部工业走廊、沈北地区和棋盘山风景区四大发展空间，城市新一轮的发展格局已经形成。

　　城市道路交通网络不断完善。地铁1、2号线全面开工建设。城区路桥改造基本完成，形成了较为快速、便捷的市区交通网络。新建农村公路4000公里，构建起覆盖全市所有行政村的农村公路网。城市道路建设开始向四大发展空间和沈阳经济区拓展，沈彰高速公路和沈抚、沈铁、苏桃等一级公路竣工通车，沈康、铁阜等高速公路开工建设，区域中心城市现代化立体交通体系基本形成。

　　奥体中心等一批标志性公共服务设施投入使用。金廊建设全面展开，一批投资百亿元以上的重大项目开工建设。城市供水、燃气、电网、通讯和防灾等公共设施日臻完善。

　　生态建设取得重大成就。浑河、蒲河、细河、满堂河、丁香湖等重点水系得到进一步治理，三环内水面积率达到7.7%。通过开展大规模绿化活动，建成区绿地率提高到38%。环境保护力度不断加大，城市污水和垃圾无害化处理率分别达到75%和100%。积极推广节能环保新技术，地源热泵应用走在全国前列。城市环境空气质量优良天数已连续4年保持在300天以上。

　　沈阳经济区建设取得重大进展。交通、环保等基础设施建设全面展开，沈抚同城化重点项目陆续启动。近海经济区建设，增强了沈阳作为连接沿海与腹地枢纽城市的聚集和辐射作用。

　　五年间，全市城乡生态环境质量发生历史性变化，沈阳实现了从工业污染城市到“国家环保模范城市”、“国家森林城市”的跨越，碧水蓝天、绿树通衢已经从梦想变成美好的现实。

　　人民生活水平和质量显著提高。2007年全市投入民生工程178.1亿元，比上年增长25.4%，占全市财政总支出的50%以上，是历年来投入最多的一年。五年来，全市对民生投入力度不断加大，累计投入532亿元，使人民群众得到更多实惠。城乡居民收入较快增长，2007年城市居民人均可支配收入15年来首次超过全国平均水平，农民人均纯收入增幅创11年来新高，人均住房建筑面积达到26.4平方米，比2002年增加7.3平方米，家庭财产增多。

　　实施了积极就业政策，就业再就业工作成效显著。2007年全市实名制就业27万人。五年共开发就业岗位56.5万个，积极稳妥地解决了国有企业27.6万“并轨”职工再就业问题，零就业家庭至少一人稳定就业，基本实现经济发展与扩大就业的良性互动。

　　社会保障体系日趋完善，保障能力不断增强。养老、职工医疗、失业、工伤、生育保险参保面逐年扩大，待遇水平逐年提高。农民工医疗保险、城镇居民基本医疗保险启动实施，新型农村合作医疗制度全面推行。

　　城乡居民最低生活保障制度不断健全。在全国率先实行了城市低保边缘户专项救助。近两年，市财政投入33亿元，较大幅度提高了以城乡低收入者为主的十方面社会群体的保障标准，惠及全市130余万人。2007年扩大了义务教育“两免一补”扶助范围，享受政策学生达到33万人。廉租住房保障制度进一步健全。群众基本生活必需品市场保障体系不断完善，强化了市场监测和调控，建立了储备制度，稳定了基本生活必需品市场，对贫困群体实施了阶段性救助。

　　社会治安防控体系日益健全，严打整治斗争取得明显成效，市民安全感不断增强。信访渠道更加畅通，群众利益诉求、表达和维护机制初步建立，社会秩序保持稳定。安全生产形势趋于平稳。公共安全管理体制进一步完善。 2007年军民携手并肩，取得了抗击特大暴风雪的胜利，经受住了城市突发事件应急处置的严峻考验。

　　近年来，市政府坚持把解决好人民群众最关心、最直接、最现实的利益问题摆在突出位置，切实为城乡居民排忧解难，做到每年办20件实事。同时，下决心解决了一大批事关群众切身利益的历史遗留问题，切实维护了人民群众的合法权益。去年，我市当选“中国十大最具幸福感城市”，是全国人民对沈阳全面振兴的高度认同，也是全市人民享有振兴成果、感受幸福的共同心声。

　　社会事业全面蓬勃发展。五年来，在加快经济发展的同时，积极推进社会建设，各项事业取得全面进步。

　　在全国率先实施义务教育均衡发展。对城区106所薄弱学校实施改造，新建农村九年一贯制学校80所，基本普及15年教育。职业教育规模不断扩大，万人规模的装备制造学校、金融学校已建成使用。沈阳职业技术学院进入国家示范性高等职业院校行列。

　　公共卫生服务体系建设进一步加强。对市属6所重点医院进行改造建设，建成310个社区卫生服务机构，改善了256家农村卫生机构医疗条件，健全了社区卫生服务和农村三级医疗服务网络，完善了突发公共卫生事件应急处置机制，有效防控和抗击了非典、禽流感等重大疫情。

　　文化事业更加繁荣。广泛开展覆盖城乡的群众文化活动，成功举办了第六届东亚儿童艺术节、中外大学校长论坛和东北文化产业博览交易会。文化精品不断涌现，文化产业有了较大发展。建设了图书馆、金融博物馆等一批文化设施。历史文化名城和文物保护工作力度加大，“一宫两陵”成功列入《世界文化遗产名录》。

　　以迎奥运为契机，全民体育健身活动掀起新的高潮，奥运足球测试赛取得成功。“迎奥运、讲文明、树新风”等群众性精神文明创建活动深入开展，市民文明素质明显提高。

　　全面推进依法行政，法治政府建设取得明显成效。行政执法监察和效能监察力度加大，创新绩效管理，强化行政责任追究，勤政廉政建设扎实推进。

　　和谐社区建设取得新进展，全市创建了115个省级先进社区。四县（市）108个村建立了村级公共服务中心，村委会建设进一步加强。

　　此外，人口与计划生育、统计、审计、物价、参事、民族、宗教、广播、电视、新闻、出版、侨务、质量技术监督、食品药品监督、气象、民兵、预备役、双拥、防震减灾、人防、档案、修志、文史、残联、红十字会和老龄工作也都取得了新成绩。

　　各位代表，过去的五年，是全市经济社会又好又快发展，城市综合实力大幅提升的五年；是沈阳国有企业改革取得历史性突破，社会主义市场经济体制日趋完善的五年；是开放的沈阳加速走向世界，城市影响力日益扩大的五年；是城市面貌发生巨大变化，区域中心城市功能逐步健全的五年；是人民群众得到实惠最多，生活质量明显改善的五年；是全市人民追求卓越、开拓进取，不断创造辉煌的五年。沈阳正在向令人瞩目、充满活力的现代化大都市昂首迈进！

　　五年来各方面成就的取得，是省委、省政府和市委正确领导的结果，得益于市人大、政协的监督和支持，凝聚着全市人民的辛劳和智慧。在此，我代表市政府，向为沈阳发展作出无私奉献、付出辛勤劳动的全市各族人民致以亲切问候和崇高敬意！向给予政府工作积极支持的人大代表、政协委员，各民主党派、工商联和无党派人士、各人民团体，向参与沈阳发展建设的中央和省在沈单位、驻沈部队指战员、武警官兵和在沈工作的外籍人士、港澳台同胞、海外侨胞及国际友人表示衷心感谢！

　　在看到成绩的同时，我们也清醒地认识到，目前我市经济和社会发展还面临着一些突出的困难和问题。一是城市综合实力不强，经济社会环境发展还不够协调；二是经济发展方式尚未根本转变，自主创新能力不强，节能减排压力较大；三是涉及人民群众切身利益的问题还比较突出，部分低收入群众生活还很困难；四是政府职能有待进一步转变，一些政府部门服务意识不强、工作作风不实、办事效率不高的现象依然存在。我们一定要高度重视，采取有力措施，切实加以解决。

　　二、今后五年经济社会发展主要目标和基本要求

　　今后五年，沈阳正处于工业化、现代化的关键阶段，也是实现老工业基地全面振兴的重要时期。党的十七大对全面振兴老工业基地提出了新的更高要求，我们必须适应国内外形势的新变化，顺应全市人民过上更好生活的新期待，坚持把沈阳的发展放在经济全球化和全国发展大格局中重新审视，抓住机遇，乘势而上，在新的历史起点上实现新的跨越。

　　今后五年政府工作的指导思想是：认真贯彻党的十七大精神，深入落实科学发展观，紧紧围绕实现“十一五”时期“三大目标”，以改革开放和自主创新为动力，着力转变经济发展方式，优化产业结构，继续保持经济又好又快发展的强劲势头；以保障、改善民生为重点，全面推进社会建设；坚持城乡统筹，促进经济社会环境协调可持续发展，开创全面建成小康社会新局面。

　　今后五年全市经济社会发展的主要目标是：在优化结构、提高效益、降低消耗、保护环境基础上，实现人均地区生产总值比二○○○年翻两番，城市综合实力进入全国十强行列。

　　根据上述指导思想和主要目标，对今后五年经济社会发展的基本要求是：

　　——推进产业结构优化升级，保持经济又好又快发展。着力打造具有国际竞争力的世界级装备制造业基地。加快推进铁西先进装备制造业聚集区建设，重点发展高档数控机床、通用机械、重矿设备、输变电等重大成套设备，培育集研发、设计、制造、安装和售后服务为一体的工程总承包公司。加快推进沈阳航空经济区规划建设，大力发展航空制造业，建设民用支线飞机研发、设计和总装基地。加快推进东部汽车产业集群建设，提高配套能力，着力打造产出千亿元的汽车整车及零部件产业集群。高水平建设公共研发、基础制造、现代物流、金融服务、人才培养等公共平台。力求在主机控制系统、动力系统、关键元器件的研发、制造和产业化上取得重大突破。鼓励装备制造骨干企业实行跨区域、跨行业、跨所有制战略重组，培育世界级装备制造业旗舰企业。

　　初步形成以服务经济为主导的产业结构。服务业占地区生产总值比重提高3个百分点以上，大力发展现代服务业，现代服务业增加值占服务业比重提高到60%。强化金融商贸开发区聚集、辐射功能，中心城区要建成以服务外包、网络信息、创意设计等领域为重点的现代服务业聚集区。加快建设东北金融、现代物流中心。

　　加快建设创新型城市。坚持创新驱动，增强自主创新能力，为转变经济发展方式、推进产业结构优化提供技术支撑。形成以企业为主体、市场为导向、产学研相结合的科技创新体系。大力引进创新型人才，构建区域人才智力高地。加快实施重大科技专项，积极抢占信息、高端制造技术制高点，加快软件、IC装备、动漫、数字医疗、数字识别等国家级产业基地建设。科技综合实力和国际竞争力明显提高。

　　全面推进县域经济发展。坚持把发展现代农业、繁荣农村经济作为社会主义新农村建设的首要任务，形成以现代农业为基础，以工业为主导，以城镇化为支撑，一、二、三产业协调发展的县域经济发展新格局。努力缩小城乡差距，基本实现县（市）郊区化，郊区城市化。

　　——着力改善民生，促进社会和谐。加快推进社会建设，全面建设覆盖城乡居民的社会服务体系，使全市人民学有所教、劳有所得、病有所医、老有所养、住有所居，共享振兴发展成果。

　　优先发展教育。全面促进义务教育均衡优质发展，普及高中教育，大力发展职业教育，建成全国一流技能人才培训基地。重视学前教育，关心特殊教育，发展社区教育，鼓励和规范社会力量兴办教育。支持在沈高校建设和发展。

　　推动文化大发展大繁荣。深化文化体制改革，积极发展公益性文化事业，加强社区和乡村文化设施建设。大力发展文化产业，加大历史文化名城保护力度，繁荣艺术精品创作。

　　健全基本医疗卫生制度。坚持公共医疗卫生的公益性质，加快建设覆盖城乡居民的公共卫生、医疗服务体系。加强医疗卫生基础设施建设，推进医疗科技发展，建设东北区域医疗服务中心。提高重大疾病防控和突发公共卫生事件应急处置能力。

　　加快建设覆盖城乡居民的社会保障体系。完善社会保险制度，逐步提高保障水平。健全城乡居民最低生活保障制度，保障困难群众基本生活。进一步完善专项救助制度，推进社会福利和慈善事业发展。

　　促进以创业带动就业。实施积极的就业政策，鼓励新增劳动力自谋职业和自主创业，健全就业援助制度，加强人力资源市场规范管理，健全统筹城乡的就业服务体系，构建和谐的劳动关系。

　　提高城乡居民收入水平。深化收入分配制度改革，着力提高低收入者收入，逐步提高最低工资标准，建立企业职工工资正常增长机制和支付保障机制，维护劳动者工资分配权益。创造条件让更多群众拥有财产性收入，积极拓展农民增收渠道，确保城乡居民收入持续稳定增加。

　　切实加强社会管理。完善信访制度，重视社会组织建设和管理，强化安全生产管理和监督，完善突发事件应急管理机制，健全社会治安防控体系，努力建设平安沈阳，全力维护社会稳定。

　　——创建国家生态市，实现可持续发展。转变增长方式，基本形成节约能源资源和保护生态环境的产业结构。全面淘汰重污染行业落后生产能力，严格限制高耗能、高排放行业的发展。“三废”和工业废物综合利用率明显提高，循环经济形成较大规模，地源热泵等节能环保新技术普遍推广，风能、太阳能、沼气等可再生能源比重显著上升。增强市民节能环保意识，基本形成文明的消费模式。提高能源资源利用效率，主要污染物排放得到有效控制。

　　加快农村环境设施建设，实施生态保护工程，扩大森林覆盖面积，推进防风固沙，农村生活和发展环境明显改善。

　　加快城市绿化和水系建设，城市水面积率达到10%以上，建成区绿化覆盖率达到42%以上，城市环境空气质量优良天数保持在320天以上，全面提高城市生态环境质量。

　　三、2008年政府主要工作

　　2008年是全面贯彻落实党的十七大精神的第一年，也是新一届政府的开局之年。

　　今年政府工作的总体要求是：深入贯彻落实科学发展观，以“优化结构年”活动为主线，坚持稳中求进、好字优先、改革开放、以人为本，加快转变经济发展方式，注重提高自主创新能力，更加重视改善民生，推动经济社会又好又快发展。

　　全市经济社会发展主要预期目标是：地区生产总值增长16%，地方财政一般预算收入增长18%，全社会固定资产投资增长16%，社会消费品零售总额增长15%，实际利用外资增长10%，城市居民人均可支配收入和农民人均纯收入分别增长13%，居民消费价格涨幅控制在4.5%以内，城镇登记失业率控制在4%以内，单位地区生产总值能耗下降4.5%，主要污染物排放总量减少3%。

　　（一）着力提高工业整体发展水平。以重特大项目建设为突破口，加快建设重点工业基地和产业集群，切实增强骨干企业发展能力，推进产业升级。规模以上工业增加值增长21%，工业固定资产投资完成950亿元。

　　突出抓好铁西装备制造业聚集区建设。重点培育15个世界级产品，做大做强装备制造业骨干企业，组建2个以重点工程、重大项目为依托的工程总承包公司。重点发展特高压输变电、盾构机、大型乙烯装置等重大成套设备, 努力在高档数控系统研发及产业化、轨道交通、风电、农机设备等领域实现突破。基本完成公共检测和物流服务平台建设。

　　抓好重特大项目开发建设。加快金新林浆纸一体化等25个已开工重大项目建设步伐，年内确保7个项目建成投产；积极推进大唐国际IGCC等10个在谈重大项目开工建设；新开工投资3000万元以上工业项目500个。加快产业集群建设，法库陶瓷等4个特色产业集群力争实现百亿产出。

　　加快航空、通讯、化工等十大重点基地规划建设，大力发展航空制造业，推进地铁装备产业化。加快汽车产业发展，年内整车产量达到42万辆。

　　（二）优先发展现代服务业。科学制定规划，加大扶持力度，全面推动服务业发展，服务业增加值增长15%。

　　改善金融发展环境，推进区域资本市场建设，拓宽企业融资渠道，提高直接融资能力，积极推动企业在境内外资本市场上市，引进外资银行3家，积极支持盛京银行在全国重点地区设立分行。加快发展银行、证券、信托、保险、基金、租赁等现代金融业。大力支持金融商贸开发区建设。

　　推进近海物流港等10个重点物流项目建设，全面提升中储等20个专业物流中心水平，引进15家国内外知名物流企业，加快地区物流总部建设。

　　大力发展软件开发、网络经营等信息服务业，发展服务外包，加快建设国家级服务外包基地。支持动漫、传媒等文化创意产业发展。积极发展会计、评估、法律服务、会展等商务服务业。

　　加快发展旅游业，精心策划并办好“世界奥林匹克城市博览会”等大型活动，建设环沈阳旅游圈，创建全国旅游最佳城市。

　　引导房地产业健康有序发展，调整住房供给结构，增加中小户型住房供应，推动房地产二级市场建设，进一步稳定房价。加快发展设计、开发、建筑、装饰、物业、交易等房地产服务业。

　　加快太原街、中街、长江街、东城、南塔等都市商贸中心改造建设，新建改造10个万米以上批发市场，大力发展社区服务业。

　　（三）提高自主创新能力。实施科技创新重点工程，着力突破制约经济社会发展的关键技术，加强新兴产业和创新型企业培育，提高科技综合实力。

　　完善高新技术产业带规划建设布局，加快中科院创新产业园、国家级大学科技园等20个高新技术产业示范区建设。浑南新区要发挥示范带头作用，重点抓好华硕电脑、力晶芯片等一批项目，推进高新技术产品出口基地建设。支持沈北新区光电通讯产业园建设，推动光电、通讯产业发展。全市孵化器面积达到270万平方米，科技投融资机构达到55家，新增科技领航型企业10家，引进研发团队60个。积极争取高档数控系统、IC装备等国家重大科技专项，加快培育和发展能源装备等十大产业。完善企业创新体系，建设20个公共研发平台，推进20个工程技术中心和重点实验室建设，抓好沈鼓集团等10家产学研联盟示范，促进工业机器人等五大产业化基地发展。

　　（四）扎实推进社会主义新农村建设。要把进一步解决好“三农”问题作为全面振兴沈阳老工业基地的重中之重，按照城乡一体化新格局的要求，加大支农惠农政策扶持力度，加快推进县域经济发展，努力增加农民收入。县域地区生产总值增长17%，地方财政一般预算收入增长30%。

　　加速发展县域工业。引进投资500万元以上工业项目800个，新上投资1000万元以上农产品加工项目150个，农产品加工率达到55%。积极推进产业集群建设，培育一批产值20亿元以上的企业集团。

　　优化农业内部结构。新增富民小区500个，“一村一业”专业村200个。加快13个农业经济区建设，积极壮大20个农业主导产业区。继续推进现代农业发展，创建农产品优质品牌，扩大高效特色农业产业规模，种植面积增长30%。

　　加大农村基础设施建设力度。新建、改造500公里中心村村内路网，建设改造500公里农村公路。实现县（市）、郊区客运公交化，全市农村行政村全部开通客运班线，新建10个农村客运站和300个农村客运候车廊。建成2个经济区垃圾处理厂，新建农村自来水工程83处，解决14万农村人口饮水安全问题。

　　加强农民实用技术培训，新增农村劳动力转移输出5万人。提高农民生产和经营组织化程度，健全城乡一体化农产品经营服务网络。

　　（五）把深化改革放在更加突出位置。全面推进重点领域和关键环节的改革。继续优化国有经济布局，推动国有资本向关键领域集中。深化国有企业股份制改造，支持沈鼓、北方重工、东北制药等大型骨干企业引进战略投资者。引进市场竞争机制，积极稳妥地推进公交、供暖、供水、供气等公用事业改革。加快地铁建设和运营的合资合作。深化国有资产管理体制改革，强化大型国有企业资产监管，逐步将事业单位国有资产全部纳入国资监管体系。毫不动摇地鼓励、支持、引导非公有制经济发展，大力发展以现代产权制度为基础的混合所有制经济，支持远大等非公有制骨干企业做大做强。

　　深化行政管理体制改革。进一步优化政府机构设置，努力解决机构重叠、职能交叉、权责脱节、政出多门的问题，提高行政效率。精简和规范各类议事协调机构及其办事机构。加快推进事业单位分类改革。

　　积极推进医药卫生体制改革，开展公立医院改革试点。推进集体林权制度改革，建立土地承包经营权流转市场。进一步调整完善市、县、区财政体制，强化预算管理，调整优化财政支出结构，加大对公共服务领域投入，重点向改善民生倾斜。

　　（六）努力构建对外开放新优势。拓展对外开放的广度和深度，把“引进来”和“走出去”结合起来，全面提高对外开放水平和质量。

　　坚持全方位对外开放，优化利用外资结构。以世界500强企业和跨国公司为重点，围绕先进装备制造、现代服务、高新技术等优先发展产业招商引资，引进超千万美元重大外资项目50个以上，引进投资500万元以上内资项目1800个。

　　加快辽宁沈阳出口加工区、沈阳（张士）出口加工区、沈阳保税物流园区建设，鼓励高科技产品和名牌产品出口，培育新的出口增长点。

　　创新对外投资和合作方式，鼓励企业开展对外工程承包，培育我市的跨国公司和知名品牌。

　　扩大对外交往，邀请更多外国政要来沈访问，积极开辟国际新航线。

　　高起点、高水平搭建对外交流合作平台，继续办好韩国周、制博会、东北亚区域发展与合作论坛、中国国际风险投资论坛等大型国际活动。

　　加强开放型经济软硬环境建设，积极筹建国际学校、国际医院等公共服务设施，形成浓厚的开放氛围。

　　（七）切实加大节能减排工作力度。把节能减排作为促进科学发展的重要抓手，采取有效措施，务求取得实效。

　　突出抓好重点行业、重点领域节能减排工作。继续抓好年耗能3000吨标煤以上企业的节能降耗工作，淘汰一批高耗能、高污染的落后产能企业，抓好沈海热电等5家电厂节能减排工程。继续实施拆除燃煤锅炉工程，对全市大型热源企业100台供暖锅炉实施脱硫治理。严格执行新建项目节能评估审查制度，控制高耗能项目建设，对高耗能、重污染企业强制实施能源审核和清洁生产审计。

　　推动建筑节能工作。全面推广保温苯板等建筑节能新材料，新建工程严格执行国家节能标准，推广城市照明节电。

　　大力推广地源热泵、中水回用等节能环保新技术，新增地源热泵应用面积1600万平方米，完成康平电厂中水回用工程。加大煤层气开发利用，全面推广清洁能源。

　　认真落实工作责任制，把完成节能减排目标作为检验经济发展成效的重要标准，实行严格的问责制和一票否决制。

　　（八）更加注重改善民生。推进城乡教育协调发展。完成城区106所薄弱学校综合改革，新建20所农村九年一贯制学校，6所万人职业学校年内全部投入使用，改善14所特殊教育学校办学条件。加快推进“双高普九”，扩大义务教育“两免一补”扶助范围，免除城市义务教育阶段学生杂费。

　　发展医疗卫生事业。大力推进6所重点医院建设，确保市第四人民医院等4所医院年内建成交付使用，其余2所医院完成主体工程建设。进一步完善城乡卫生服务网络，在有条件的城区推行社区卫生服务“收支两条线，药品零差价”管理，成立市紧急医疗救援中心，为城乡居民提供安全、方便、价廉的基本医疗服务。

　　促进公益性文化事业发展，不断壮大传媒、演艺等经营性文化产业。加强社区和乡村文化设施建设。认真做好历史文化遗产保护，对“一宫两陵帅府”实施内部修缮和外部环境综合整治，建设和改造东北近代历史博物馆等3座博物馆，加快棋盘山国家级文化创意产业示范区建设。

　　进一步做好就业再就业工作。全市新增就业岗位10万个。开展普惠制就业培训，加大对大龄失业、被征地农民、零就业家庭等困难群体就业援助力度。完善创业服务体系和政策体系，促进以创业带动就业。做好高校毕业生、退伍转业军人安置工作。做好农民工工资支付保障工作，切实维护农民工合法权益。

　　加快完善社会保障体系。继续做好社会保险扩面工作，切实加强社会保险基金征收和监管，确保企业基本养老金按时足额发放，探索建立农村养老保险制度。解决好困难企业职工参加基本医疗保险问题，积极推进城镇居民基本医疗保险，新型农村合作医疗人均筹资标准提高到120元。加强非公有制企业从业人员、灵活就业人员、被征地农民社会保障工作。不断健全社会救助体系。建立农村低保边缘户救助制度，加强对城乡困难群体医疗、教育、供暖等专项救助，做好“两节”临时救济。发展残疾人、老人等社会福利事业，开展慈善援助活动。

　　今年，要以救助困难群体和特殊群体为重点，新增投资24亿元，实施7项帮扶任务。全年投入106亿元，全面提高城乡低保户、低保边缘户、企业退休职工、农村五保户等十一类群体的保障救助标准，惠及全市123万人。

　　采取多种方式解决城乡困难群体住房问题。新建5万平方米廉租住房，解决1000户城市低保家庭住房困难，新增115万平方米经济适用住房，改善2万户城市低收入家庭居住条件。帮助1000户农村贫困家庭解决住房。

　　设立粮食、肉类等基本生活必需品市场保障资金，进一步完善监测预警机制、储备机制、应急调控机制，平抑物价，稳定市场，加大对困难群体的救助力度。

　　加强城乡社区建设，完善社区组织网络和服务功能。强化社会治安综合治理，严厉打击刑事犯罪和经济犯罪活动，努力提高刑事案件破案率，严防重大刑事案件发生。进一步做好安全生产工作，有效遏制重特大安全事故，增强人民群众安全感。

　　深入开展双拥工作，巩固双拥成果，努力争取全国双拥模范七连冠。

　　今年继续为城乡群众办好20件实事。

　　（九）全面提高城市建设和管理水平。要以城市核心区和大浑南地区为重点，全面推进城市基础设施、社会公共设施和生态环境重大项目建设。全年城建投资280亿元。

　　加快奥体中心三馆建设，开工建设艺术中心。加快推进地铁1、2号线建设。沈康、铁阜、辽宁中部环线辽阳至新民段等高速公路年底前通车。

　　完善三环内道路交通网络。改造关键交通节点，加快五爱隧道、南八马路、黄河立交桥等重点工程改造建设，切实解决城区主干道交通拥堵问题。建设西站、五爱综合交通换乘枢纽。三环内建成区入住2000户以上的小区全部通公交。加强中心城区与四大发展空间的交通联系，启动西北开发大道建设。

　　加强水系、绿化建设。以南运河改造为重点，抓好截污活水、美化亮化等工程，实施浑河浑南拦河坝改造，加快百里环城水系建设，全市新增水面积3.4平方公里。重点搞好二环三环沿线、社区和街路绿化，在金廊沿线等街路建设10条林荫大道，新增绿地面积6平方公里。建设南部污水处理厂、工业园区及县域污水处理厂，提高城市污水处理能力。

　　加大金廊重点项目建设力度。金廊中部和南部要形成规模、早见形象，北部金廊要加快建设。

　　适应城市发展需要，加快三环内和大浑南地区电力、供暖、燃气、给排水等市政基础设施配套建设。

　　切实加强城市规划，强化土地依法管理，集约利用土地，规范和完善土地交易市场。

　　以迎奥运为契机，提高城市管理水平。推行城市数字化管理，做好重点街路亮化、绿化、净化，加强市容环境综合治理。加强药品、食品安全综合监管，完善突发事件应急管理机制，确保2008奥运足球比赛取得圆满成功。

　　加快沈阳经济区建设。启动沈本产业大道建设，推进沈铁、沈本城际客运公交化，进一步开展经贸、环保、旅游等领域合作，沈抚同城化规划建设要取得实质进展。

　　（十）努力建设服务型政府。健全向人大常委会报告工作机制，自觉接受人大法律监督和工作监督。主动接受政协的民主监督。认真办理人大代表的议案、建议和政协委员的提案。

　　进一步转变政府职能。加快推进政企分开、政资分开、政事分开、政府与中介组织分开，要规范行政行为，坚持用制度管权、管事、管人，巩固、扩大行政审批制度改革成果。

　　坚持依法行政，加强政府立法工作，强化行政监督，做到严格执法、公正执法和文明执法，提高政府执行力和公信力。

　　完善公共服务体系，推行电子政务，强化社会管理和公共服务职能。推行政务公开，不断提高政府工作透明度。畅通政府与群众沟通渠道，切实保障人民群众知情权、参与权、监督权。

　　加强公务员队伍建设，严格公务员管理，提高公务员行政能力。加强对权力的制约，特别是对领导干部、人财物管理使用、关键岗位的监督和制约，加大对滥用权力和失职渎职行为的追究力度。

　　各级政府要切实转变思想作风、领导作风和工作作风。要树立正确的政绩观，多干打基础、利长远的事，力戒急于求成、急功近利。加强调查研究，精简会议和文件，反对形式主义，反对弄虚作假，以求真务实作风推进各项工作。坚持全心全意为人民服务，真诚倾听群众呼声，真实反映群众愿望，真情关心群众疾苦，多为群众办好事、办实事。

　　各位代表，实现沈阳未来五年发展的美好蓝图,是时代赋予我们的神圣使命，也是740万沈阳人民的共同期盼。站在新的历史起点上，让我们高举中国特色社会主义伟大旗帜，深入贯彻落实科学发展观，励精图治，奋发进取，为实现沈阳老工业基地振兴，全面建成小康社会而努力奋斗！
